# The Psychological Impact of Foreign Assignments on Diplomats and Their Families: A Systematic Review

**DOI:** 10.1192/j.eurpsy.2026.10742

**Published:** 2026-07-31

**Authors:** Z. Malaniuková, M. Anders

**Affiliations:** 1st faculty of medicine, Charles University, Prague, Czech Republic

## Abstract

**Introduction:**

Diplomatic personnel and their families face unique psychosocial stressors, including frequent relocations, limited autonomy, and deployment to high-risk environments. Despite potential psychiatric consequences, systematic evidence remains scarce.

**Objectives:**

To synthesise empirical findings on mental health outcomes, occupational stressors, coping resources, and family experiences in order to inform institutional support and policy.

**Methods:**

Systematic searches of Web of Science and Scopus (April 2024) were conducted following PRISMA 2020 guidelines. Eligible studies included empirical, peer-reviewed research addressing the psychological wellbeing of diplomats or their families. Six studies met inclusion criteria. Given methodological heterogeneity, findings were synthesised narratively.

**Results:**

Stressors identified included heavy workload, constrained autonomy, relocations, and deployment to hardship or conflict zones. Outcomes encompassed elevated stress, burnout, and reduced quality of life, with higher PTSD symptoms in conflict postings. Protective factors comprised social support, self-efficacy, and dual national identification. Spouses reported emotional strain, isolation, and insufficient institutional recognition, particularly during the COVID-19 pandemic.

**Conclusions:**

Diplomatic service entails specific psychological vulnerabilities. Evidence highlights the importance of psychosocial preparation, family-inclusive policies, and culturally responsive support. Future studies should adopt longitudinal designs and evaluate targeted interventions to improve wellbeing in foreign service populations.

**Disclosure of Interest:**

None Declared

